# Crystal structure of 2,4-bis­(2-chloro­phen­yl)-7-*tert*-pent­yl-3-aza­bicyclo[3.3.1]nonan-9-one

**DOI:** 10.1107/S160053681402176X

**Published:** 2014-10-15

**Authors:** Dong Ho Park, V. Ramkumar, P. Parthiban

**Affiliations:** a Department of Biomedicinal Chemistry, Inje University, Gimhae, Gyeongnam 621 749, Republic of Korea; b Department of Chemistry, IIT Madras, Chennai 600 036, TamilNadu, India; c Department of Chemistry, VEL TECH, Avadi, Chennai 600 062, India

**Keywords:** crystal structure, twin-chair conformation, Mannich base, aza­bicycle

## Abstract

The title compound, C_25_H_29_Cl_2_NO, which is a chloro analog of 2,4-bis­(2-bromo­phen­yl)-7-(*tert*-pent­yl)-3-aza­bicyclo­[3.3.1]nonan-9-one [Park, Ramkumar & Parthiban (2012). *Acta Cryst.* E**68**, o2946], exists in a twin-chair conformation with an equatorial orientation of the 2-chloro­phenyl groups. The *tert*-pentyl group on the cyclo­hexa­none adopts an exocyclic equatorial position and is disordered between two orientations in a ratio 0.520 (8):0.480 (8). The crystal packing shows no directional contacts beyond van der Waals contacts.

## Related literature   

For the synthesis, stereochemistry and biological activity of 3-aza­bicyclo­[3.3.1]nonan-9-ones, see: Park *et al.* (2011 ▶, 2012*a* ▶). For a related crystal structure, see: Park *et al.* (2012*b* ▶). For the conformation of functionalized 3-aza­bicycles, see: Parthiban *et al.* (2010 ▶); Park *et al.* (2012*c* ▶); Padegimas & Kovacic (1972 ▶).
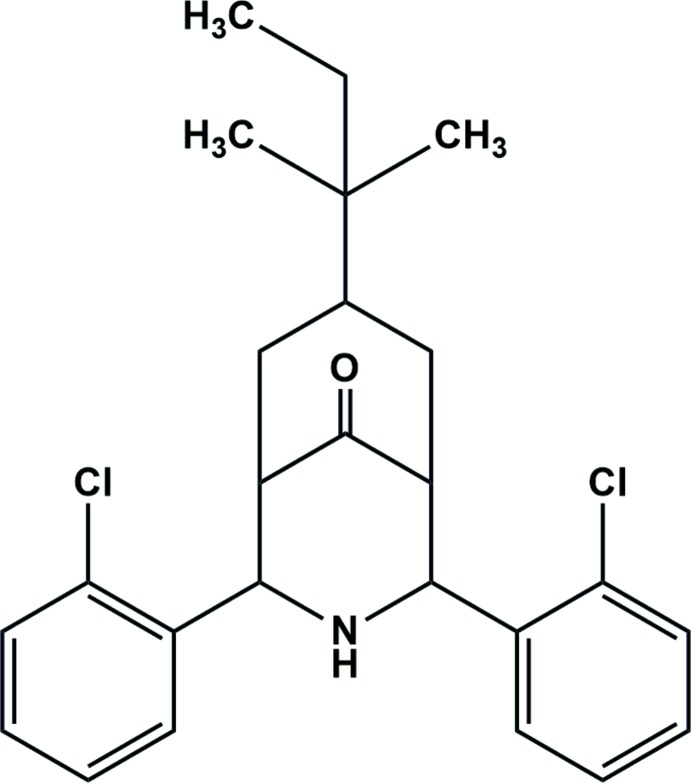


## Experimental   

### Crystal data  

C_25_H_29_Cl_2_NO
*M* *_r_* = 430.39
Triclinic, 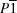

*a* = 7.6006 (3) Å
*b* = 10.6240 (5) Å
*c* = 15.1124 (7) Å
α = 106.116 (2)°
β = 99.996 (2)°
γ = 98.266 (2)°
*V* = 1130.54 (9) Å^3^
*Z* = 2
Mo *K*α radiation
μ = 0.30 mm^−1^
*T* = 298 K
0.25 × 0.20 × 0.15 mm

### Data collection  

Bruker APEXII area-detector diffractometer
Absorption correction: multi-scan (*SADABS*; Bruker, 2004 ▶) *T*_min_ = 0.928, *T*_max_ = 0.955
13160 measured reflections
3789 independent reflections
2944 reflections with *I* > 2σ(*I*)
*R*_int_ = 0.020

### Refinement  

*R*[*F* ^2^ > 2σ(*F* ^2^)] = 0.042
*wR*(*F* ^2^) = 0.119
*S* = 1.03
3789 reflections
313 parameters
13 restraints
H atoms treated by a mixture of independent and constrained refinement
Δρ_max_ = 0.36 e Å^−3^
Δρ_min_ = −0.27 e Å^−3^

### 

Data collection: *APEX2* (Bruker, 2004 ▶); cell refinement: *APEX2* and *SAINT-Plus* (Bruker, 2004 ▶); data reduction: *SAINT-Plus* and *XPREP* (Bruker, 2004 ▶); program(s) used to solve structure: *SHELXS97* (Sheldrick, 2008 ▶); program(s) used to refine structure: *SHELXL2013* (Sheldrick, 2008 ▶); molecular graphics: *ORTEP-3 for Windows* (Farrugia, 2012 ▶); software used to prepare material for publication: *SHELXL2013*.

## Supplementary Material

Crystal structure: contains datablock(s) global, I. DOI: 10.1107/S160053681402176X/cv5472sup1.cif

Structure factors: contains datablock(s) I. DOI: 10.1107/S160053681402176X/cv5472Isup2.hkl

Supporting information file. DOI: 10.1107/S160053681402176X/cv5472Isup3.cml

**Fig. 1.**
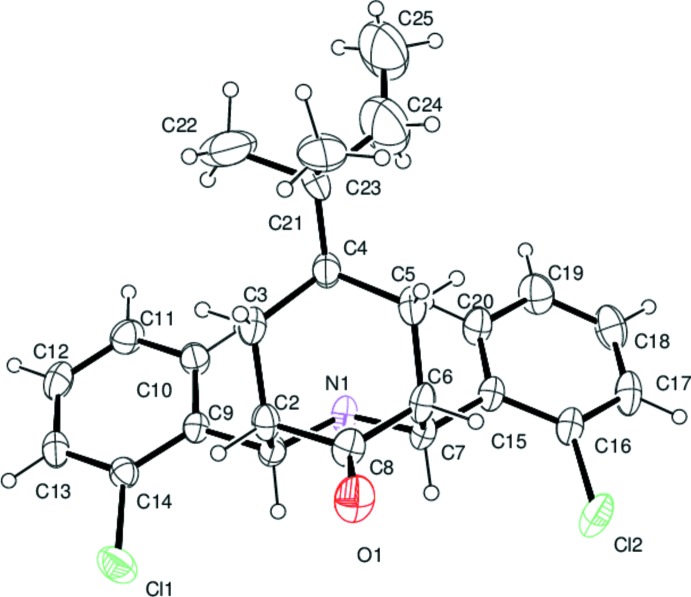
View of the title molecule showing the atomic numbering and 30% probability displacement ellipsoids. For clarity, only major component of the disordered group is shown.

. DOI: 10.1107/S160053681402176X/cv5472fig1.tif
View of the title mol­ecule showing the atomic numbering and 30% probability displacement ellipsoids. For clarity, only major component of the disordered group is shown.

CCDC reference: 1027325

Additional supporting information:  crystallographic information; 3D view; checkCIF report

## supplementary crystallographic information

### S1. Comment

Three major conformations, *viz*., chair-chair (Parthiban *et al.*, 2010), chair-boat (Park *et al.*, 2012*c*), and boat-boat (Padegimas & Kovacic, 1972) are possible for the bicycle. Hence, the present study is to investigate the stereochemistry of the title compound.

The detailed analysis of asymmetry parameters and torsion angles of the title compound reveal that the values are very similar to its bromo analog. The torsion angles of the title compound C5—C6—C8—C2, C3—C2—C8—C6, C7—C6—C8—C2 and C1—C2—C8—C6 are 63.0 (2), -63.0 (2), -62.6 (2) and 62.4 (2)°, respectively. These values indicate the slightly distorted chair conformation for both six-membered cycles of the fused bicycle.

The orientation of the chlorophenyl groups on both sides of the secondary amino group is identified as equatorial by their torsion angles. The torsion angle of C15—C7—C6—C8 and C8—C2—C1—C9 are 179.99 (17) and -179.01 (17)°, respectively. The orientation of *tert*-pentyl group on the cyclohexanone ring is also identified as equatorial by the following torsion angles: C21—C4—C5—C6 and C21—C4—C3—C2 are 172.3 (9) and -172.6 (12)°, respectively [C21A—C4—C5—C6 and C21A—C4—C3—C2 are 173.7 (10) and -172.9 (11)°, respectively].

The chloro substituted benzene rings of the title compound is oriented very similar to that of its bromo analog. The benzene rings are inclined to each other with an angle of 29.38°, where as the orientation of bromo analog is 29.6 (3)°.

Based on the complete crystallographic analysis, it is concluded that the title compound, C_25_H_29_Cl_2_NO, exists in a twin-chair conformation with an equatorial orientation of the 2-chlorophenyl groups.

### S2. Experimental

2,4-*Bis*(2-chlorophenyl)-7-(*tert*-pentyl)-3-azabicyclo[3.3.1]nonan-9-one was synthesized by a modified and an optimized Mannich condensation in one-pot, using 2-chlorobenzaldehyde (0.1 mol, 14.06 g/11.25 ml), 4-*tert*-pentylcyclohexanone (0.05 mol, 8.41 g/9.15 ml) and ammonium acetate (0.075 mol, 5.78 g) in a 50 ml of absolute ethanol (Park *et al.*, 2011). The mixture was gently warmed on a hot plate at 303–308 K (30–35° C) with moderate stirring till the complete consumption of the starting materials, which was monitored by TLC. At the end, the crude azabicyclic ketone was separated by filtration and gently washed with 1:5 cold ethanol-ether mixture. X-ray diffraction quality crystals of the title compound were obtained by slow evaporation from ethanol.

### S3. Refinement

All hydrogen atoms were fixed geometrically and allowed to ride on the parent carbon atoms with aromatic C—H = 0.93 Å, aliphatic C—H = 0.98 Å, methylene C—H = 0.97 Å. The displacement parameters were set for phenyl, methylene and aliphatic H atoms at *U*_iso_(H) = 1.2*U*_eq_(C), methyl H atoms at *U*_iso_(H) = 1.5*U*_eq_(C) and the hydrogen atoms were fixed geometrically and allowed to ride on the parent nitrogen atom with N—H = 0.86 Å and the displacement parameter was set at *U*_iso_(H)= 1.2*U*_eq_(N). The *tert*-pentyl group attached to the carbon atom (C4) is disordered in two orientations in a ratio 0.520 (8):0.480 (8).

### Crystal data

**Table:**

C_25_H_29_Cl_2_NO	*V* = 1130.54 (9) Å^3^
*M**_r_* = 430.39	*Z* = 2
Triclinic, *P*1	*F*(000) = 456
*a* = 7.6006 (3) Å	*D*_x_ = 1.264 Mg m^−^^3^
*b* = 10.6240 (5) Å	Mo *K*α radiation, λ = 0.71073 Å
*c* = 15.1124 (7) Å	µ = 0.30 mm^−^^1^
α = 106.116 (2)°	*T* = 298 K
β = 99.996 (2)°	Block, colourless
γ = 98.266 (2)°	0.25 × 0.20 × 0.15 mm

### Data collection

**Table:**

Bruker APEXII area-detector diffractometer	2944 reflections with *I* > 2σ(*I*)
phi and ω scans	*R*_int_ = 0.020
Absorption correction: multi-scan (*SADABS*; Bruker, 2004)	θ_max_ = 25.0°, θ_min_ = 2.0°
*T*_min_ = 0.928, *T*_max_ = 0.955	*h* = −9→6
13160 measured reflections	*k* = −12→12
3789 independent reflections	*l* = −17→17

### Refinement

**Table:**

Refinement on *F*^2^	13 restraints
Least-squares matrix: full	Hydrogen site location: mixed
*R*[*F*^2^ > 2σ(*F*^2^)] = 0.042	H atoms treated by a mixture of independent and constrained refinement
*wR*(*F*^2^) = 0.119	*w* = 1/[σ^2^(*F*_o_^2^) + (0.0537*P*)^2^ + 0.5471*P*] where *P* = (*F*_o_^2^ + 2*F*_c_^2^)/3
*S* = 1.03	(Δ/σ)_max_ = 0.041
3789 reflections	Δρ_max_ = 0.36 e Å^−^^3^
313 parameters	Δρ_min_ = −0.27 e Å^−^^3^

### Special details

**Table:**

Geometry. All e.s.d.'s (except the e.s.d. in the dihedral angle between two l.s. planes) are estimated using the full covariance matrix. The cell e.s.d.'s are taken into account individually in the estimation of e.s.d.'s in distances, angles and torsion angles; correlations between e.s.d.'s in cell parameters are only used when they are defined by crystal symmetry. An approximate (isotropic) treatment of cell e.s.d.'s is used for estimating e.s.d.'s involving l.s. planes.

### Fractional atomic coordinates and isotropic or equivalent isotropic displacement parameters (Å^2^)

**Table:**

	*x*	*y*	*z*	*U*_iso_*/*U*_eq_	Occ. (<1)
C1	0.3313 (3)	0.57407 (19)	0.12669 (15)	0.0406 (5)	
H1	0.2254	0.5266	0.0749	0.049*	
C2	0.2630 (3)	0.6552 (2)	0.21189 (16)	0.0442 (5)	
H2	0.1886	0.7138	0.1906	0.053*	
C3	0.4123 (3)	0.7409 (2)	0.29862 (15)	0.0468 (5)	
H3A	0.4952	0.8002	0.2787	0.056*	
H3B	0.3556	0.7964	0.3432	0.056*	
C4	0.5240 (3)	0.6624 (2)	0.35002 (16)	0.0466 (5)	
H4	0.6008	0.6236	0.3083	0.056*	
C5	0.3990 (3)	0.5446 (2)	0.36240 (16)	0.0486 (6)	
H5A	0.3413	0.5789	0.4140	0.058*	
H5B	0.4737	0.4851	0.3804	0.058*	
C6	0.2491 (3)	0.4627 (2)	0.27445 (16)	0.0471 (5)	
H6	0.1661	0.3997	0.2925	0.057*	
C7	0.3167 (3)	0.38288 (19)	0.18868 (15)	0.0428 (5)	
H7	0.2097	0.3330	0.1382	0.051*	
C8	0.1434 (3)	0.5563 (2)	0.24180 (16)	0.0488 (6)	
C9	0.4562 (3)	0.66577 (19)	0.09259 (14)	0.0380 (5)	
C10	0.6447 (3)	0.6856 (2)	0.12042 (16)	0.0465 (5)	
H10	0.6951	0.6381	0.1580	0.056*	
C11	0.7590 (3)	0.7744 (2)	0.09362 (18)	0.0562 (6)	
H11	0.8847	0.7862	0.1138	0.067*	
C12	0.6891 (4)	0.8452 (2)	0.03757 (17)	0.0573 (6)	
H12	0.7669	0.9052	0.0201	0.069*	
C13	0.5037 (3)	0.8269 (2)	0.00741 (15)	0.0494 (6)	
H13	0.4550	0.8737	−0.0312	0.059*	
C14	0.3896 (3)	0.7384 (2)	0.03483 (14)	0.0419 (5)	
C15	0.4261 (3)	0.28405 (19)	0.21254 (15)	0.0425 (5)	
C16	0.3442 (3)	0.1555 (2)	0.20782 (16)	0.0488 (6)	
C17	0.4439 (4)	0.0637 (2)	0.22588 (19)	0.0623 (7)	
H17	0.3849	−0.0216	0.2213	0.075*	
C18	0.6297 (4)	0.0981 (3)	0.2506 (2)	0.0695 (8)	
H18	0.6979	0.0362	0.2625	0.083*	
C19	0.7157 (4)	0.2251 (3)	0.2577 (2)	0.0665 (7)	
H19	0.8423	0.2494	0.2755	0.080*	
C20	0.6147 (3)	0.3163 (2)	0.23847 (17)	0.0532 (6)	
H20	0.6748	0.4013	0.2430	0.064*	
C21	0.647 (3)	0.7469 (17)	0.4380 (15)	0.072 (6)	0.520 (8)
C22	0.753 (3)	0.8725 (19)	0.4286 (14)	0.124 (8)	0.520 (8)
H22A	0.7762	0.8544	0.3663	0.186*	0.520 (8)
H22B	0.8667	0.9015	0.4745	0.186*	0.520 (8)
H22C	0.6831	0.9416	0.4389	0.186*	0.520 (8)
C23	0.565 (3)	0.805 (3)	0.5161 (11)	0.105 (7)	0.520 (8)
H23A	0.6564	0.8702	0.5660	0.157*	0.520 (8)
H23B	0.5143	0.7356	0.5393	0.157*	0.520 (8)
H23C	0.4701	0.8471	0.4944	0.157*	0.520 (8)
C24	0.777 (3)	0.668 (2)	0.4817 (17)	0.171 (5)	0.520 (8)
H24A	0.7085	0.6296	0.5198	0.205*	0.520 (8)
H24B	0.7801	0.5927	0.4283	0.205*	0.520 (8)
C25	0.9450 (13)	0.7029 (12)	0.5319 (9)	0.171 (5)	0.520 (8)
H25A	1.0225	0.7434	0.4993	0.256*	0.520 (8)
H25B	0.9861	0.6252	0.5413	0.256*	0.520 (8)
H25C	0.9494	0.7659	0.5922	0.256*	0.520 (8)
C21A	0.665 (4)	0.754 (2)	0.4492 (15)	0.076 (6)	0.480 (8)
C22A	0.799 (2)	0.6604 (15)	0.4763 (10)	0.142 (8)	0.480 (8)
H22D	0.8658	0.7005	0.5407	0.212*	0.480 (8)
H22E	0.8830	0.6503	0.4353	0.212*	0.480 (8)
H22F	0.7287	0.5741	0.4692	0.212*	0.480 (8)
C23A	0.544 (4)	0.820 (3)	0.5231 (16)	0.136 (10)	0.480 (8)
H23D	0.4741	0.8750	0.4969	0.204*	0.480 (8)
H23E	0.6245	0.8743	0.5820	0.204*	0.480 (8)
H23F	0.4636	0.7503	0.5335	0.204*	0.480 (8)
C24A	0.784 (3)	0.8687 (16)	0.4277 (14)	0.084 (5)	0.480 (8)
H24C	0.7014	0.9185	0.4028	0.101*	0.480 (8)
H24D	0.8568	0.9285	0.4879	0.101*	0.480 (8)
C25A	0.8920 (15)	0.8455 (10)	0.3726 (7)	0.128 (4)	0.480 (8)
H25D	0.9835	0.8035	0.3980	0.192*	0.480 (8)
H25E	0.9496	0.9282	0.3664	0.192*	0.480 (8)
H25F	0.8247	0.7873	0.3116	0.192*	0.480 (8)
Cl2	0.10719 (9)	0.10441 (6)	0.17580 (6)	0.0745 (2)	
Cl1	0.15563 (9)	0.72003 (8)	−0.00583 (5)	0.0713 (2)	
N1	0.4263 (2)	0.47552 (17)	0.15359 (13)	0.0433 (4)	
O1	−0.0167 (2)	0.55371 (18)	0.24000 (15)	0.0717 (5)	
H1N	0.461 (3)	0.428 (2)	0.1038 (17)	0.054 (7)*	

### Atomic displacement parameters (Å^2^)

**Table:**

	*U*^11^	*U*^22^	*U*^33^	*U*^12^	*U*^13^	*U*^23^
C1	0.0404 (11)	0.0351 (10)	0.0469 (12)	0.0060 (9)	0.0096 (9)	0.0147 (9)
C2	0.0461 (12)	0.0391 (11)	0.0580 (13)	0.0172 (10)	0.0191 (10)	0.0231 (10)
C3	0.0616 (14)	0.0324 (10)	0.0516 (13)	0.0109 (10)	0.0202 (11)	0.0157 (10)
C4	0.0573 (14)	0.0387 (11)	0.0478 (13)	0.0085 (10)	0.0142 (11)	0.0192 (10)
C5	0.0654 (15)	0.0404 (11)	0.0503 (13)	0.0143 (11)	0.0229 (11)	0.0224 (10)
C6	0.0508 (13)	0.0365 (11)	0.0656 (14)	0.0079 (10)	0.0296 (11)	0.0245 (10)
C7	0.0455 (12)	0.0306 (10)	0.0536 (13)	0.0040 (9)	0.0142 (10)	0.0151 (9)
C8	0.0461 (14)	0.0454 (12)	0.0600 (14)	0.0121 (10)	0.0226 (11)	0.0168 (11)
C9	0.0432 (12)	0.0318 (10)	0.0406 (11)	0.0083 (9)	0.0120 (9)	0.0119 (9)
C10	0.0440 (13)	0.0477 (12)	0.0535 (13)	0.0115 (10)	0.0130 (10)	0.0226 (10)
C11	0.0452 (13)	0.0586 (14)	0.0659 (15)	0.0032 (11)	0.0173 (12)	0.0217 (13)
C12	0.0693 (17)	0.0440 (12)	0.0609 (15)	−0.0005 (12)	0.0261 (13)	0.0192 (11)
C13	0.0719 (17)	0.0372 (11)	0.0455 (12)	0.0144 (11)	0.0191 (12)	0.0176 (10)
C14	0.0481 (12)	0.0381 (11)	0.0405 (11)	0.0124 (9)	0.0105 (9)	0.0117 (9)
C15	0.0519 (13)	0.0318 (10)	0.0481 (12)	0.0084 (9)	0.0195 (10)	0.0141 (9)
C16	0.0623 (14)	0.0350 (11)	0.0536 (13)	0.0080 (10)	0.0231 (11)	0.0160 (10)
C17	0.092 (2)	0.0373 (12)	0.0723 (17)	0.0202 (13)	0.0353 (15)	0.0262 (12)
C18	0.090 (2)	0.0608 (16)	0.0817 (19)	0.0423 (16)	0.0340 (16)	0.0373 (14)
C19	0.0578 (16)	0.0714 (17)	0.0844 (19)	0.0266 (14)	0.0247 (14)	0.0345 (15)
C20	0.0545 (15)	0.0424 (12)	0.0703 (16)	0.0131 (11)	0.0219 (12)	0.0231 (11)
C21	0.088 (9)	0.066 (9)	0.071 (9)	0.014 (8)	−0.001 (6)	0.048 (8)
C22	0.109 (12)	0.121 (12)	0.091 (10)	−0.032 (8)	−0.022 (8)	0.006 (8)
C23	0.157 (13)	0.077 (7)	0.044 (7)	0.004 (9)	−0.017 (9)	−0.004 (6)
C24	0.107 (6)	0.190 (8)	0.238 (11)	0.020 (6)	−0.016 (6)	0.142 (8)
C25	0.107 (6)	0.190 (8)	0.238 (11)	0.020 (6)	−0.016 (6)	0.142 (8)
C21A	0.088 (10)	0.059 (8)	0.044 (6)	−0.039 (7)	−0.007 (6)	0.001 (5)
C22A	0.150 (12)	0.108 (7)	0.114 (7)	−0.078 (8)	−0.109 (8)	0.088 (6)
C23A	0.219 (19)	0.100 (14)	0.074 (9)	−0.004 (12)	0.078 (11)	−0.003 (8)
C24A	0.073 (7)	0.076 (8)	0.111 (11)	−0.020 (5)	0.004 (6)	0.069 (8)
C25A	0.135 (9)	0.100 (6)	0.122 (8)	0.002 (6)	0.007 (7)	0.015 (6)
Cl2	0.0669 (4)	0.0464 (3)	0.1071 (6)	−0.0064 (3)	0.0240 (4)	0.0251 (3)
Cl1	0.0531 (4)	0.0934 (5)	0.0785 (5)	0.0225 (4)	0.0065 (3)	0.0456 (4)
N1	0.0528 (11)	0.0326 (9)	0.0536 (11)	0.0125 (8)	0.0256 (9)	0.0176 (8)
O1	0.0477 (10)	0.0753 (12)	0.1089 (15)	0.0197 (9)	0.0349 (10)	0.0412 (11)

### Geometric parameters (Å, º)

**Table:**

C1—N1	1.461 (3)	C17—C18	1.364 (4)
C1—C9	1.516 (3)	C17—H17	0.9300
C1—C2	1.554 (3)	C18—C19	1.378 (4)
C1—H1	0.9800	C18—H18	0.9300
C2—C8	1.505 (3)	C19—C20	1.380 (3)
C2—C3	1.536 (3)	C19—H19	0.9300
C2—H2	0.9800	C20—H20	0.9300
C3—C4	1.534 (3)	C21—C23	1.46 (3)
C3—H3A	0.9700	C21—C22	1.51 (3)
C3—H3B	0.9700	C21—C24	1.56 (3)
C4—C21	1.454 (19)	C22—H22A	0.9600
C4—C5	1.535 (3)	C22—H22B	0.9600
C4—C21A	1.636 (19)	C22—H22C	0.9600
C4—H4	0.9800	C23—H23A	0.9600
C5—C6	1.539 (3)	C23—H23B	0.9600
C5—H5A	0.9700	C23—H23C	0.9600
C5—H5B	0.9700	C24—C25	1.309 (18)
C6—C8	1.497 (3)	C24—H24A	0.9700
C6—C7	1.552 (3)	C24—H24B	0.9700
C6—H6	0.9800	C25—H25A	0.9600
C7—N1	1.467 (3)	C25—H25B	0.9600
C7—C15	1.511 (3)	C25—H25C	0.9600
C7—H7	0.9800	C21A—C24A	1.55 (3)
C8—O1	1.209 (3)	C21A—C22A	1.61 (3)
C9—C10	1.388 (3)	C21A—C23A	1.64 (4)
C9—C14	1.395 (3)	C22A—H22D	0.9600
C10—C11	1.381 (3)	C22A—H22E	0.9600
C10—H10	0.9300	C22A—H22F	0.9600
C11—C12	1.370 (4)	C23A—H23D	0.9600
C11—H11	0.9300	C23A—H23E	0.9600
C12—C13	1.371 (3)	C23A—H23F	0.9600
C12—H12	0.9300	C24A—C25A	1.27 (3)
C13—C14	1.384 (3)	C24A—H24C	0.9700
C13—H13	0.9300	C24A—H24D	0.9700
C14—Cl1	1.742 (2)	C25A—H25D	0.9600
C15—C20	1.384 (3)	C25A—H25E	0.9600
C15—C16	1.394 (3)	C25A—H25F	0.9600
C16—C17	1.374 (3)	N1—H1N	0.89 (2)
C16—Cl2	1.745 (2)		
			
N1—C1—C9	110.16 (16)	C16—C17—H17	120.1
N1—C1—C2	109.81 (17)	C17—C18—C19	119.6 (2)
C9—C1—C2	111.03 (16)	C17—C18—H18	120.2
N1—C1—H1	108.6	C19—C18—H18	120.2
C9—C1—H1	108.6	C18—C19—C20	120.3 (3)
C2—C1—H1	108.6	C18—C19—H19	119.9
C8—C2—C3	108.14 (18)	C20—C19—H19	119.9
C8—C2—C1	107.25 (16)	C19—C20—C15	121.5 (2)
C3—C2—C1	115.74 (18)	C19—C20—H20	119.3
C8—C2—H2	108.5	C15—C20—H20	119.3
C3—C2—H2	108.5	C4—C21—C23	117.1 (18)
C1—C2—H2	108.5	C4—C21—C22	113.0 (14)
C4—C3—C2	115.32 (17)	C23—C21—C22	100.3 (18)
C4—C3—H3A	108.4	C4—C21—C24	111.8 (14)
C2—C3—H3A	108.4	C23—C21—C24	102.4 (18)
C4—C3—H3B	108.4	C22—C21—C24	111 (2)
C2—C3—H3B	108.4	C21—C22—H22A	109.4
H3A—C3—H3B	107.5	C21—C22—H22B	109.5
C21—C4—C3	113.1 (6)	H22A—C22—H22B	109.5
C21—C4—C5	112.9 (9)	C21—C22—H22C	109.5
C3—C4—C5	110.78 (19)	H22A—C22—H22C	109.5
C3—C4—C21A	114.5 (8)	H22B—C22—H22C	109.5
C5—C4—C21A	112.2 (9)	C21—C23—H23A	109.5
C21—C4—H4	106.5	C21—C23—H23B	109.5
C3—C4—H4	106.5	H23A—C23—H23B	109.5
C5—C4—H4	106.5	C21—C23—H23C	109.4
C4—C5—C6	115.05 (18)	H23A—C23—H23C	109.5
C4—C5—H5A	108.5	H23B—C23—H23C	109.5
C6—C5—H5A	108.5	C25—C24—C21	132.5 (17)
C4—C5—H5B	108.5	C25—C24—H24A	103.9
C6—C5—H5B	108.5	C21—C24—H24A	103.9
H5A—C5—H5B	107.5	C25—C24—H24B	104.4
C8—C6—C5	108.55 (17)	C21—C24—H24B	104.3
C8—C6—C7	107.09 (18)	H24A—C24—H24B	105.5
C5—C6—C7	115.73 (18)	C24—C25—H25A	109.3
C8—C6—H6	108.4	C24—C25—H25B	109.3
C5—C6—H6	108.4	H25A—C25—H25B	109.5
C7—C6—H6	108.4	C24—C25—H25C	109.8
N1—C7—C15	109.85 (17)	H25A—C25—H25C	109.5
N1—C7—C6	109.86 (16)	H25B—C25—H25C	109.5
C15—C7—C6	112.34 (18)	C24A—C21A—C22A	106 (2)
N1—C7—H7	108.2	C24A—C21A—C4	107.5 (15)
C15—C7—H7	108.2	C22A—C21A—C4	105.6 (13)
C6—C7—H7	108.2	C24A—C21A—C23A	108.6 (19)
O1—C8—C6	124.7 (2)	C22A—C21A—C23A	120.0 (18)
O1—C8—C2	124.2 (2)	C4—C21A—C23A	108.1 (18)
C6—C8—C2	111.14 (18)	C21A—C22A—H22D	109.6
C10—C9—C14	116.29 (18)	C21A—C22A—H22E	109.3
C10—C9—C1	121.34 (18)	H22D—C22A—H22E	109.5
C14—C9—C1	122.33 (18)	C21A—C22A—H22F	109.5
C11—C10—C9	121.6 (2)	H22D—C22A—H22F	109.5
C11—C10—H10	119.2	H22E—C22A—H22F	109.5
C9—C10—H10	119.2	C21A—C23A—H23D	109.5
C12—C11—C10	120.7 (2)	C21A—C23A—H23E	109.5
C12—C11—H11	119.7	H23D—C23A—H23E	109.5
C10—C11—H11	119.7	C21A—C23A—H23F	109.4
C11—C12—C13	119.5 (2)	H23D—C23A—H23F	109.5
C11—C12—H12	120.2	H23E—C23A—H23F	109.5
C13—C12—H12	120.2	C25A—C24A—C21A	121.8 (19)
C12—C13—C14	119.6 (2)	C25A—C24A—H24C	106.9
C12—C13—H13	120.2	C21A—C24A—H24C	106.9
C14—C13—H13	120.2	C25A—C24A—H24D	106.9
C13—C14—C9	122.3 (2)	C21A—C24A—H24D	106.9
C13—C14—Cl1	117.25 (17)	H24C—C24A—H24D	106.7
C9—C14—Cl1	120.43 (16)	C24A—C25A—H25D	109.5
C20—C15—C16	116.5 (2)	C24A—C25A—H25E	109.5
C20—C15—C7	121.22 (18)	H25D—C25A—H25E	109.5
C16—C15—C7	122.3 (2)	C24A—C25A—H25F	109.5
C17—C16—C15	122.3 (2)	H25D—C25A—H25F	109.5
C17—C16—Cl2	117.35 (18)	H25E—C25A—H25F	109.5
C15—C16—Cl2	120.29 (18)	C1—N1—C7	114.20 (16)
C18—C17—C16	119.8 (2)	C1—N1—H1N	108.7 (16)
C18—C17—H17	120.1	C7—N1—H1N	108.5 (15)
			
N1—C1—C2—C8	−56.9 (2)	C6—C7—C15—C20	−95.7 (2)
C9—C1—C2—C8	−179.01 (17)	N1—C7—C15—C16	−151.6 (2)
N1—C1—C2—C3	63.8 (2)	C6—C7—C15—C16	85.8 (2)
C9—C1—C2—C3	−58.2 (2)	C20—C15—C16—C17	−1.3 (3)
C8—C2—C3—C4	53.9 (2)	C7—C15—C16—C17	177.4 (2)
C1—C2—C3—C4	−66.4 (2)	C20—C15—C16—Cl2	−179.97 (17)
C2—C3—C4—C21	−172.6 (12)	C7—C15—C16—Cl2	−1.3 (3)
C2—C3—C4—C5	−44.7 (2)	C15—C16—C17—C18	0.8 (4)
C2—C3—C4—C21A	−172.9 (11)	Cl2—C16—C17—C18	179.6 (2)
C21—C4—C5—C6	172.3 (9)	C16—C17—C18—C19	0.4 (4)
C3—C4—C5—C6	44.4 (2)	C17—C18—C19—C20	−1.1 (4)
C21A—C4—C5—C6	173.7 (10)	C18—C19—C20—C15	0.6 (4)
C4—C5—C6—C8	−53.6 (2)	C16—C15—C20—C19	0.6 (3)
C4—C5—C6—C7	66.8 (2)	C7—C15—C20—C19	−178.1 (2)
C8—C6—C7—N1	57.4 (2)	C3—C4—C21—C23	70.0 (17)
C5—C6—C7—N1	−63.8 (2)	C5—C4—C21—C23	−56.8 (16)
C8—C6—C7—C15	−179.99 (17)	C21A—C4—C21—C23	−120 (81)
C5—C6—C7—C15	58.8 (2)	C3—C4—C21—C22	−46 (2)
C5—C6—C8—O1	−116.6 (3)	C5—C4—C21—C22	−172.5 (16)
C7—C6—C8—O1	117.7 (2)	C21A—C4—C21—C22	125 (82)
C5—C6—C8—C2	63.0 (2)	C3—C4—C21—C24	−172.4 (14)
C7—C6—C8—C2	−62.6 (2)	C5—C4—C21—C24	61 (2)
C3—C2—C8—O1	116.6 (3)	C21A—C4—C21—C24	−2 (79)
C1—C2—C8—O1	−117.9 (2)	C4—C21—C24—C25	148 (3)
C3—C2—C8—C6	−63.0 (2)	C23—C21—C24—C25	−86 (3)
C1—C2—C8—C6	62.4 (2)	C22—C21—C24—C25	20 (4)
N1—C1—C9—C10	−23.9 (3)	C21—C4—C21A—C24A	−63 (79)
C2—C1—C9—C10	97.9 (2)	C3—C4—C21A—C24A	−53 (2)
N1—C1—C9—C14	158.49 (18)	C5—C4—C21A—C24A	179.7 (14)
C2—C1—C9—C14	−79.6 (2)	C21—C4—C21A—C22A	−176 (100)
C14—C9—C10—C11	1.2 (3)	C3—C4—C21A—C22A	−166.3 (11)
C1—C9—C10—C11	−176.5 (2)	C5—C4—C21A—C22A	66.3 (17)
C9—C10—C11—C12	−0.6 (4)	C21—C4—C21A—C23A	54 (80)
C10—C11—C12—C13	−0.4 (4)	C3—C4—C21A—C23A	64.1 (18)
C11—C12—C13—C14	0.7 (3)	C5—C4—C21A—C23A	−63.3 (17)
C12—C13—C14—C9	−0.1 (3)	C22A—C21A—C24A—C25A	49 (2)
C12—C13—C14—Cl1	179.93 (17)	C4—C21A—C24A—C25A	−64 (2)
C10—C9—C14—C13	−0.8 (3)	C23A—C21A—C24A—C25A	179.7 (17)
C1—C9—C14—C13	176.83 (18)	C9—C1—N1—C7	179.34 (17)
C10—C9—C14—Cl1	179.14 (15)	C2—C1—N1—C7	56.8 (2)
C1—C9—C14—Cl1	−3.2 (3)	C15—C7—N1—C1	178.90 (17)
N1—C7—C15—C20	27.0 (3)	C6—C7—N1—C1	−57.0 (2)
