# Supplementary figures and images for: Crystal structure of 2,4-bis­(2-chloro­phen­yl)-7-tert-pent­yl-3-aza­bicyclo[3.3.1]nonan-9-one
Source: Acta Crystallogr Sect E Struct Rep Online. 2014 Oct 15;70(Pt 11):o1161–2. doi: 10.1107/S160053681402176X (PMC4257353; doi:10.1107/S160053681402176X)

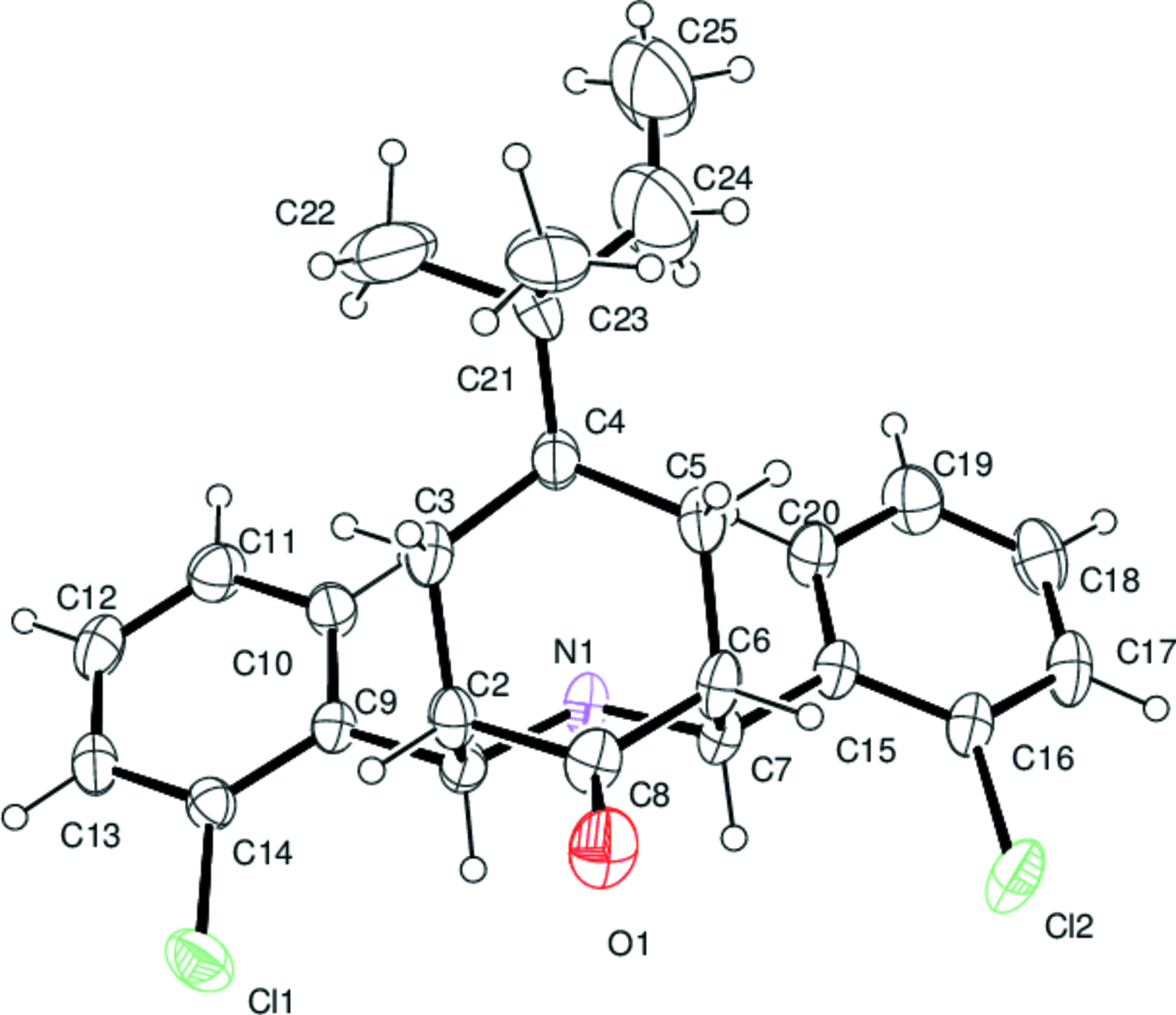

Supplement: Supplementary file 4 [file e-70-o1161-fig1.tif]
